# Synovial biopsies in clinical practice and research: current developments and perspectives

**DOI:** 10.1007/s10067-020-05512-7

**Published:** 2020-12-04

**Authors:** Hanna Johnsson, Aurélie Najm

**Affiliations:** Institute of Infection, Immunity and Inflammation, College of Medical Veterinary and Life Sciences, University of Glasgow and Rheumatology Department Greater Glasgow and Clyde, Glasgow, UK

**Keywords:** Arthritis, Biopsy, Synovial biopsy, Synovitis, Synovium, Ultrasound

## Abstract

Synovial biopsy techniques have developed and widely expanded over the past few years, in particular due to the development of ultrasound-guided procedures. This article reviews the different techniques, clinical applications, and the latest advances in translational research as well as current challenges and perspectives. The first part focuses on different techniques available for biopsy, along with their feasibility, success rate, tolerance, and training requirements. In the second part, clinical applications are described. Data on diagnostic performances are reported, especially regarding septic arthritis. Translational research applications are described and explained in the final part, from the early histological studies and the first description of pathotype to more recent technologies involving -omics. Latest developments involving single-cell RNA sequence analysis have allowed the discovery of new cell subpopulations with remarkable roles in RA pathophysiology. These studies pave the ground for the discovery of new therapeutic targets and the implementation of personalized therapy in RA.

**Table:** 

**Key Point** **•***This review provides an overview of synovial biopsy techinques and applications especially in clinical and translational research.*

**Key Point**

**•***This review provides an overview of synovial biopsy techinques and applications especially in clinical and translational research.*

## Different biopsy techniques

### Brief history and introduction of techniques

Arthroscopic synovial biopsy has traditionally been considered the gold standard for obtaining synovial tissue. It enables the operator to view the synovium macroscopically and choose where to take the biopsy [1]. The macroscopic inspection can also yield diagnostic information [2]. With the invention of thinner arthroscopes, the procedure became less invasive, and more rheumatologists performed arthroscopic biopsies throughout the 1990s [3].

However, arthroscopic synovial biopsies are invasive, expensive, and not widely available. Alongside the practice of arthroscopic synovial biopsy procedures, less invasive, simpler, and cheaper techniques for obtaining synovial biopsies were developed. Early blind biopsy needles measured 5 mm across so remained invasive [4]. Subsequent blind biopsy needles, like the Parker and Pearson needle, were thinner (14G, 15G, and 18G) and widely used [5, 6]. Samples obtained from the suprapatellar pouch by blind needle biopsies show broadly similar features to samples taken using arthroscopy [4, 7], but sufficient tissue for histological analysis is only obtained in 61 to 85% of cases [8, 9]. The procedure is also limited to larger joints.

To improve success rates and ensure that biopsies are taken from the synovium, imaging-guided biopsy techniques evolved. Imaging modalities reported include fluoroscopy and CT scanning [10, 11], but ultrasound (US) has become the preferred method to visualize the joint. US is free of ionizing radiation, and many rheumatologists use US in their daily clinical practice.

**Fig. 1:**
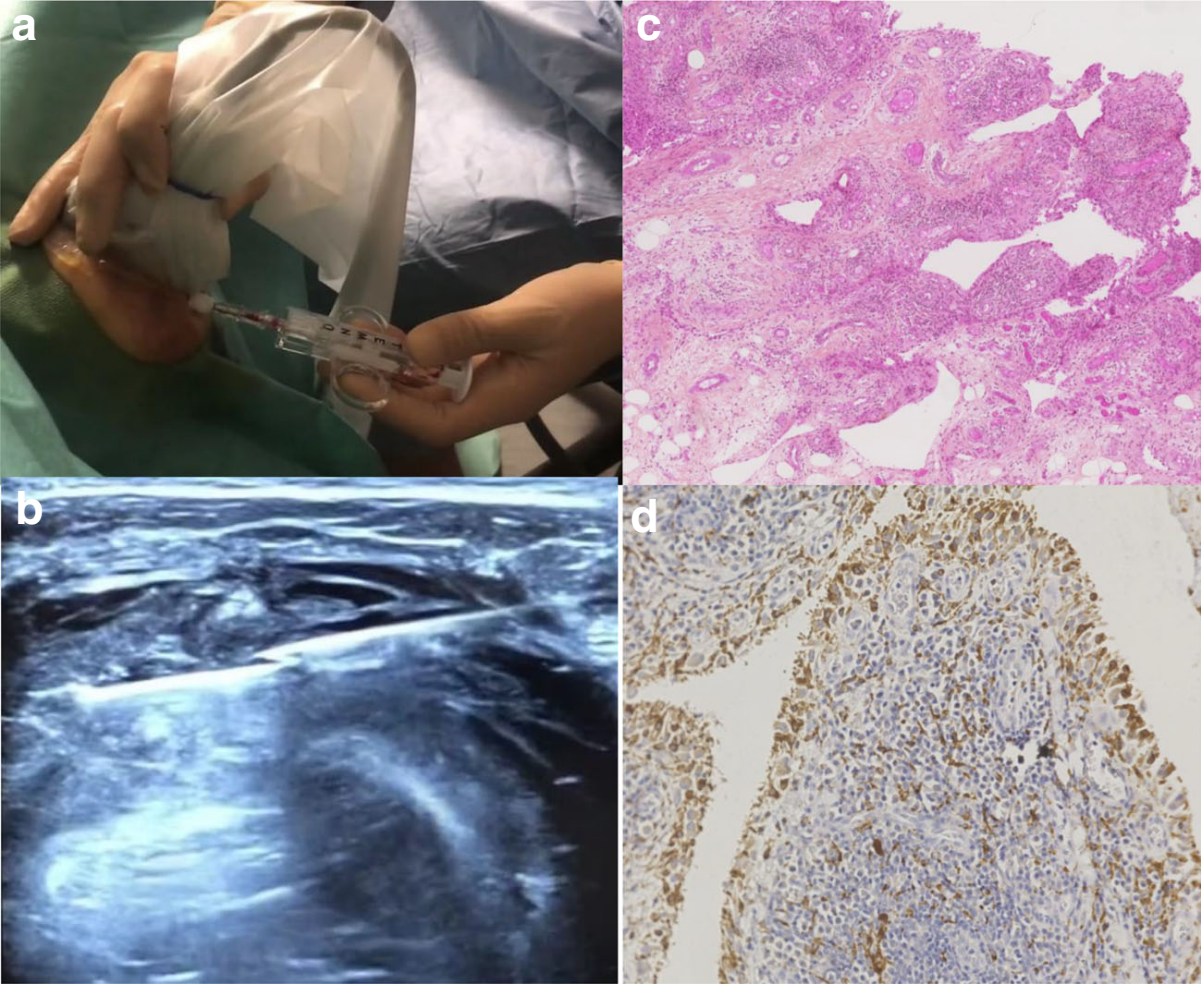
**a** and **b** Ultrasound guided synovial biopsy. **a** Procedure with the semi-automatic guillotine needle. **b** US image of a shoulder synovitis and the biopsy needle. **c** and **d** Synovial tissue from an inflamed knee joint in RA. **c** Haematoxylin and Eosin staining. **d** CD68+ macrophages staining

Since first reported in 1997 [12], US-guided synovial biopsies (US-SB) are now routinely performed in many centres throughout the world. An advantage of US-SB over arthroscopic and blind biopsies is that they can safely and successfully be performed on both large and small joints [13–15]. The most frequently biopsied joints are wrists, knees, MCPs, and ankles, but also elbows, shoulders, hips, sternoclavicular, acromioclavicular, and pubic symphysis biopsies are performed [13, 15, 16]. Figure 1 shows photos of an US-SB being performed, and the microscopic appearances of the tissue obtained.

### What is involved for the patient?

Arthroscopic synovial biopsies are typically performed in an operating theatre or in a procedure room [3]. Most are performed using local anaesthetic, but general anaesthetic is used in more than 1 in 5 procedures [16]. Half of the centres always or often prescribe sedation [3]. Two ports are required: one for the arthroscope and one for the biopsy forceps. The diameter of the arthroscope is between 1.9 and 4.5 mm [6]. The operator can inspect the macroscopic appearance of the joint and choose the biopsy site. Adequate samples can be obtained also after inflammation has been successfully treated [6]. Joint lavage with saline can be performed, and this can be therapeutic [1]. The port sites are usually closed with steristrips, though sutures are sometimes used [3]. No specific observation is required for most procedures.

US-SB require less specialist equipment than arthroscopic biopsies. They can be obtained using portal and biopsy forceps or a semi-automated core biopsy needle. In both cases, local anaesthetic is infiltrated into the skin, soft tissues, and synovial space. For portal and forceps, the portal is inserted into the synovial space under US guidance and biopsy forceps introduced through the portal. Semi-automated core biopsy needles can either be introduced repeatedly through the same tract under US guidance or through an introducer which acts as a portal. US grey scale and power Doppler signal guide the operator to the site of the highest inflammation. Multiple synovial biopsies are taken, ideally from different parts of the joint. As the puncture site usually is no larger than 2 mm, steri strips are sufficient to close the wound. The procedure typically takes less than 1 h to perform, and patients can leave soon thereafter. Patients are advised to rest the biopsied joint for 48 h after the procedure.

### Patient acceptance and tolerability

Minimally invasive synovial biopsy procedures are well-tolerated. A comparison of 402 US-guided needle biopsies, 65 US-guided portal and forceps biopsies, and 57 arthroscopic-guided synovial biopsies did not identify any difference in the post-biopsy pain, swelling, or stiffness between the three procedures [16]. Pain, swelling, and stiffness are unchanged or reduced following biopsy compared to before the procedure [16–18]. Steroids reduce post-procedure swelling significantly but do not improve pain or stiffness significantly [16]. The discomfort during the procedure is higher for large joints than small- and medium-sized joints [17].

The majority of patients is somewhat or very likely to agree to another biopsy, with patient undergoing US-guided needle biopsies less willing than those undergoing US-guided portal and forceps biopsies or arthroscopic biopsies [16–18]. The willingness does not decline after a second biopsy [16].

### Complications

The complication rates of minimally invasive arthroscopic and US-SB are similar between studies and summarized in Table 1.

**Table 1:** Success rate, complications, and tolerability of arthroscopic and US-guided synovial biopsies [1, 3, 13–21]

	Arthroscopic	US-guided biopsy semi-automatic needle	US-guided biopsy portal and forceps
Success rate	> 90%	Around 80–90% depends on US grey scale synovitis grade, joint size, and operator experience	Around 80–90%
Complications	1.51% complication rate, haemarthrosis 0.9%, DVT 0.2%, wound infection 0.1%, joint infection 16 0.1%, thrombophlebitis 0.08%, flare of gout 0.06%, syncope/vasovagal 0.02%, neurological 0.02%	Minor bleeding up to 7.8%, haemarthrosis 0.3–1.3%, neurological up to 3.1%, vasovagal symptoms 0.8 to 3.2%, rare: tenosynovitis	Skin infection (1 of 37 procedures in one study)
Tolerability	62% no or mild discomfort	70–92.8% no or mild discomfort	71% no or mild discomfort
Willingness of repeat biopsy	64% likely or somewhat likely	57–74% likely or somewhat likely	85% likely or somewhat likely

Fewer studies have reported on US-guided port-and-forceps biopsies than arthroscopic and US-guided needle biopsies

Based on a survey of 15682 procedures, the most common complications of arthroscopic synovial biopsies performed by rheumatologists are haemarthosis in 0.9% and DVT in 0.2% of patients. Joint infection, wound infection, and neurological damage occur in 0.1%, 0.1%, and 0.02%, respectively [3].

Studies on the complication rates of US-SB are smaller, and many do not report any major complications. The largest study to date included 402 US-guided needle biopsies and 65 US-guided portal and forceps biopsies and identified a complication rate of 8/467 (1.71%) [16]. These comprised of 2 episodes of syncope or presyncope, 1 tenosynovitis, 4 sensory impairment, and 1 haemarthrosis. Syncope or presyncope have been reported by others as between 0.8 and 3.2% [16, 17, 19]. Motor neurological complications, both transient paresis due to local anaesthesia and persistent mild limitation of digit extension, have occurred following wrist biopsies [18]. Minor bleeding occurs in up to 7.8% of procedures [18], but haemarthrosis is rare with rates up to 1.3% in individual studies [15]. Skin infection was reported in one study [14].

The most common minor complications following US-SB are mild arthralgia and increased use of analgesia following the procedure, occurring following 19.4% and 36% procedures, respectively [17, 18]. There is no increase in adverse event in patients on biologic treatments [17].

### Success of procedure

The definition of success varies between studies historically. Current recommendations stipulate that the synovial lining layer should be present and that an area of 2.5 mm^2^ is required for representative staining [22]. Due to tissue heterogeneity, it is recommended that at least four biopsies are taken from small joints and six biopsies from large joints [22]. The yield of tissue depends on US grey scale synovitis with the highest yield with synovial grade 3 [17]. There is a trend for more successful samples from large joints than small joints.

Of 7 studies included in a systematic review, only three clearly defined success as an intact lining layer [13]. The success rate in these three studies was 89–100%. A somewhat lower success rate of US-SB was reported in an observational study in which 62 of 76 clinical procedures (81.6%) yielded synovial tissues [15]. Synovial lining was seen in 92.6% of successful procedures. The failed biopsies occurred in both small and large joints, and the authors comment that inexperience contributed to biopsy failure. The proportion of gradable synovial tissue is lower when blind needle biopsies are used compared to US-guided and arthroscopic procedures [9]. For large joints, US-SB perform as well as arthroscopic biopsies.

### Training

Arthoscopic biopsies require considerable training. Observation of 5 procedures, assisting at 10 procedures and performing 10 procedures supervised, has been proposed as adequate training before independence [3]. More recently, the European League Against Rheumatism (EULAR) has been working towards the creation of a EULAR standardized training model for ultrasound-guided synovial biopsy procedures in large and small joints leading to an educational video and recommendations (Moller et al., ongoing).

## Clinical utility

### Synovial biopsy to exclude infection

The most common clinical indication for synovial biopsies is to rule out septic arthritis [15, 20]. In a study reporting on 76 US-guided procedures performed with clinical indications, 82.4% was performed to rule out septic arthritis [15]. Most patients had an undifferentiated chronic monoarthritis (54.1%) or an acute monoarthritis (24%), but cases of chronic undifferentiated oligoarthritis, chronic polyarthritis, chronic bursitis, chronic tenosynovitis, and acute polyarthritis were also biopsied. Two cases of infectious arthritis were identified, namely Lyme’s disease and articular Whipple disease [15]. In the case of Whipple disease, the synovial fluid PCR had been negative. No other case of infectious arthritis was identified during follow-up, suggesting that synovial biopsy is reliable in excluding septic arthritis. The reliability of synovial biopsies to exclude infection in native joints has been replicated by others [18, 19].

A causative organism is identified in less than half of patients who clinically have septic arthritis, and a synovial biopsy can be informative [20, 23]. In a study where 90% of participants had a synovial fluid aspirate and 25% had a synovial biopsy taken, the causative organism was grown in 38.7% of synovial fluid samples and 23.5% of synovial membranes. Antibiotics had not been given prior to synovial fluid aspirate but could have been given before the synovial biopsy. Nonetheless, in three cases (one mycobacterium and two *Staphylococcus aureus*), the synovial tissue was positive on culture, but the synovial fluid was negative [20, 23]. Moreover, bacterial DNA is detectable in synovial tissue for longer than gram stain and positive cultures and can be detected after empirical antibiotics have been started [24]. A high synovial neutrophil (CD15+) count (absolute and relative) is also supportive of a diagnosis of septic arthritis [20, 25]. When synovial biopsies are performed in patients with suspected infectious arthritis, qPCR analysis for 16S RNA, Borrelia Burgdorferi and Tropheryma Whipplei are recommended [22].

### Diagnoses made with synovial biopsies

In most cases, synovial biopsies taken with clinical indications do not yield a specific diagnosis [15, 18]. Nonetheless, a number of specific diagnosis have been reported, including gout and pseudogout with deposits found within the synovial tissue, even when synovial fluid analysis is negative [6, 15, 18]. Importantly, the synovial tissue needs to be preserved in absolute ethanol rather than formalin to prevent urate crystals from dissolving.

Other pathologies which can be identified on synovial biopsy include amyloidosis with deposits which stain red with Congo red stain and synovial chondromatosis with cartilage nodules seen within the synovial tissue [18, 26]. In ochronosis, haemachromatosis, and recurrent haemarthrosis, specific pigments are seen. A brown colour from macrophages carrying haemosiderin is also seen in pigmented villonodular synovitis along with hypercellularity in the sublining layer. There are rare synovial malignancies including synovial chondrosarcoma, synovial haemangiomas, lipoma aborescens, and intracapsular chondromas [26]. The pathological diagnosis of tumours identified by synovial biopsies is compatible with pathological diagnosis from synovectomy samples [19]. Also lymphoprolipherative disorders can present within the synovium, and sarcoidosis, foreign body arthritis, the histiocytic disorder Erdheim-Chester disease and multicentric reticulohistocytosis have been diagnosed with synovial biopsies [15, 18, 19, 27].

### Utility in inflammatory arthritis

In cases lacking clear distinguishing pathological features, synovial biopsies can still support a diagnosis of inflammatory arthritis. The Krenn score gives a score between 0 and 3 to the histological features of enlargement of synovial lining cell layer, density of resident cells, and inflammatory infiltrate. It defines synovitis histologically as low-grade (2–4) and high-grade (5–9) synovitis [28]. Using the cut-off of 4, inflammatory arthritis can be differentiated from healthy controls and degenerative disease with a specificity of 96.1% and sensitivity of 61.7% [28]. By adding immunostaining with CD68, CD3, CD20, CD31, and Ki67, the specificity and sensitivity for differentiating inflammatory arthritis from osteoarthritis are improved [29].

To date, there is no validated histological score which can differentiate between different types of inflammatory arthritis in individual patients. On a disease level, however, there are differences. Patients with spondyloarthritis (SpA) have increased vasculature with tortuous vessels, and this is seen both macroscopically and microscopically [1, 30]. The cellular infiltrate in the synovium is different depending on the presence of a joint effusion in RA and SpA [30]. Patients with RA with joint effusions have increased B cell and T cell staining compared to patients with SpA with joint effusions. Furthermore, patients with early RA have increased staining of plasma cells, B cells, and macrophages compared to non-RA early arthritis [31]. Minimal staining of plasma cells and macrophages predicts a diagnosis other than RA in 96% cases [31]. In addition, specific synovial signatures including the expression of chemokines ((C-X-C motif) ligand 4 (CXCL4) and CXCL7), and fibroblast activation protein (FAP) differentiate early RA from other inflammatory arthritis of recent onset [32, 33].

## Translational research

With the wider availability of US-SB, the procedure is increasingly used to obtain tissue for research. Thus, the analysis of synovial tissue has been used over the past few decades in translational research to increase our knowledge and understanding of the pathophysiology of rheumatic diseases, to predict treatment response, and to help discover new therapeutic targets.

Given the sensitivity to change of the tissue, especially in terms of cell infiltrate, synovitis histological assessment is considered a reliable biomarker of response to treatment [34].

### Histological stratification

Histological analysis, by permitting the analysis of tissue cell infiltrates in RA synovium, allows for better stratification of RA patients across disease states and phenotypes. This has led to the concept of synovial pathotypes which was first introduced by Dennis et al. [35]. Pathotypes are defined by a specific cell type enrichment within the synovial tissue and are associated with a well-defined underlying molecular signature [35]. Four different pathotypes were initially described: lymphoid enriched in B and T cells, myeloid enriched in macrophages, fibroid, and pauci-immune [35]. The concept of pathotypes was further developed by others as lympho-myeloid, diffuse-myeloid, and pauci-immune, and these three patterns are present also in early RA [21, 36].

Numerous studies have assessed the relationship between pathotypes and RA disease phenotypes. The lymphoid pathotype is associated with the presence of ectopic lymphoid follicles. Their presence in the synovial tissue has been inconsistently reported as associated with disease activity and an erosive RA phenotype [21, 37–40]. The pauci-immune phenotype is associated with lower levels of CRP [21, 41].

This being said, cell populations within the synovial membrane infiltrate are highly heterogeneous, and are influenced by many different parameters, such as disease duration [42], ACPA status in RA [43, 44], disease activity [45], and biological treatment. Therefore, studies focusing on gene expression signatures in addition to, or instead of, the histological cell composition have been developed in order to provide a more accurate patient stratification and personalized therapies approaches.

### Towards personalized therapy using molecular signatures

The histological pathotypes are associated with well-defined molecular signatures [21, 35, 46]. The inclusion of this information in prediction models can improve the sensitivity and specificity of patients’ classification in comparison to 1987 ACR criteria [47] in a cohort of 200 early arthritis patients, including RA and undifferentiated arthritis patients [46]. Myeloid- and lymphoid-associated genes correlate to disease activity and response to treatment at 6 months, while a lymphoid molecular signature correlates to an osteoclasts-related gene enrichment and predicted structural damages progression at 12 months [21].

Although histologically defined pathotypes are not associated with csDMARD response in early RA, related molecular signatures are associated with response to csDMARDs [21]. Furthermore, the lympho-myeloid pathotype, with enriched inflammatory gene expression including IL-6 and TNFα, is associated with a higher prescription of biological DMARDs [46].

Pathotypes and molecular signatures have also been used to predict response to biological treatments. For example, patients with the myeloid phenotype are better responders to TNF blockade [35], and transcripts associated with lymphoid pathotype predict a good response to infliximab therapy [48]. Enrichment of B cells in cellular infiltrates and B cell linage transcripts on the other hand are associated with a poor response to TNF inhibitors [49], as is the pauci-immune phenotype [41].

Of interest, Badot et al. performed gene expression studies along with immunohistochemistry without consideration of pathotypes [50]. They demonstrated that high baseline synovial expression of interleukin-7 receptor alpha chain (IL-7R), CXCL11, IL-18, IL-18 receptor accessory (IL-18rap), and MKI67 predicts a poor response to adalimumab therapy [50].

These studies show promise for personalized medicine in RA, and further studies are ongoing. Alongside, new single-cell and -omics technologies are rapidly improving our understanding of disease pathophysiology and may enable the identification of new therapeutic targets.

### New single-cell technologies

New distinct cell subpopulations have been identified in RA using technologies linking transcriptomics and proteomics through single-cell RNA seq, mass spectrometry, bulk RNA seq, and flow cytometry. The Accelerating Medicines Partnership Rheumatoid Arthritis and Lupus (AMP RA/SLE) consortium mapped cell subpopulations according to their production of cytokines [51]. Indeed, new T cell populations, such as PDCD1^+^ T peripheral helper and T follicular helper T cells, were newly described, and a pro-inflammatory subpopulation of sublining fibroblasts, THY1(CD90)^+^HLA-DRA^hi^, is the main producer of IL-6. The pathogenic role of resident synovial fibroblasts was also emphasized in recent work from Croft et al., who described novel fibroblast subpopulations within RA synovial tissue using single-cell RNA sequencing [52]. Two populations with distinct roles were described: the fibroblast activation protein-α (FAPα)- positive thymus cell antigen 1 (THY1) positive synovial fibroblasts (FAPα+THY1+) located in the synovial sublining and able to regulate inflammation through cytokines and chemokines secretion, and the FAPα+THY1-fibroblasts located in the lining layer responsible for bone and cartilage destruction and an invasive phenotype.

More recently, Alivernini et al. studied macrophage subpopulations across disease states in RA, using synovial tissue from active RA and remission [53]. They identified two new subpopulations of synovial tissue macrophages (MerTK^pos^CD206^pos^ and MerTK^pos^LYVE1^pos^) displaying transcriptomic signatures enriched in negative regulators of inflammation. The presence of these populations was associated with remission maintenance, suggesting a promising potential therapeutic role of these cells in RA.

Although most work has focused on RA, Wade et al. identified a polyfunctional T cell subset in PsA using flow cytometry of digested PsA synovial tissue [54]. These CD4 + CD161+ T cells produce high concentrations of pro-inflammatory cytokines, including IL-17A, IFNγ, and TNF. Their presence correlates highly to PsA disease activity expressed by the disease activity in psoriatic arthritis (DAPSA).

Further agenda in the field of synovial tissue research includes projects related to education, clinical applications, translational research, quality appraisal of research, and therapy. The research agenda is detailed in Table 2.

**Table 2:** Research agenda

Education	
○ Establishment of consensual training requirements	
○ Development of a training framework and practical opportunities	
Clinical applications	
○ Development and implementation of synovial tissue biomarkers for diagnosis or prognosis for RMDs in clinical practice	
Translational research	
○ Synovial tissue atlas (multi-omics approach) in RMDs	
○ Assessment of tissular miRNA signature through single-cell approaches (single-cell miRNome) in RMDs	
Quality appraisal of research	
○ EULAR points to consider for minimal reporting requirements in synovial tissue clinical practice and research in rheumatology	
Therapy	
○ Personalized medicine and sequential therapy in RMDs	
○ Synovial cells reprogramming	

In conclusion, RA and other rheumatic diseases are characterized by synovial inflammation, and the retrieval and analysis of synovial biopsies have improved the understanding of disease pathophysiology. Minimally invasive US-SB techniques are safe, well-tolerated, and widely performed. In addition to a demonstrated utility in different clinical situations, synovial tissue analysis, at a cellular and molecular level initially and a molecular lever more recently, has driven important developments in rheumatology. The implementation and use of cutting-edge technologies in translational research are currently allowing new insights and identification of new cell subsets involved in different states of rheumatic diseases. Such data will allow a better understanding of disease phenotypes, predict treatment response, and constitute a rationale for the development of new cell-targeted therapies.

## Data Availability

Not applicable
